# Supplementary material for: Influence of Morphometry on Echocardiographic Measurements in Cavalier King Charles Spaniels: An Inverse Probability Weighting Analysis
Source: Vet Sci. 2021 Sep 23;8(10):205. doi: 10.3390/vetsci8100205 (PMC8538534; doi:10.3390/vetsci8100205)
Supplement: Supplementary file 1 [file vetsci-08-00205-s001.zip › vetsci-1349478-supplementary.pdf]

*Article*

# Influence of Morphometry on Echocardiographic Measurements in Cavalier King Charles Spaniels: An Inverse Probability Weighting Analysis

**Mara Bagardi [1,*], Sara Ghilardi [1], Chiara Locatelli [1], Arianna Bionda [1], Michele Polli [1], Claudio M. Bussadori [2], Fabio M. Colombo [3], Laura Pazzagli [4] and Paola G. Brambilla [1]**

**Supplementary Figure S1**

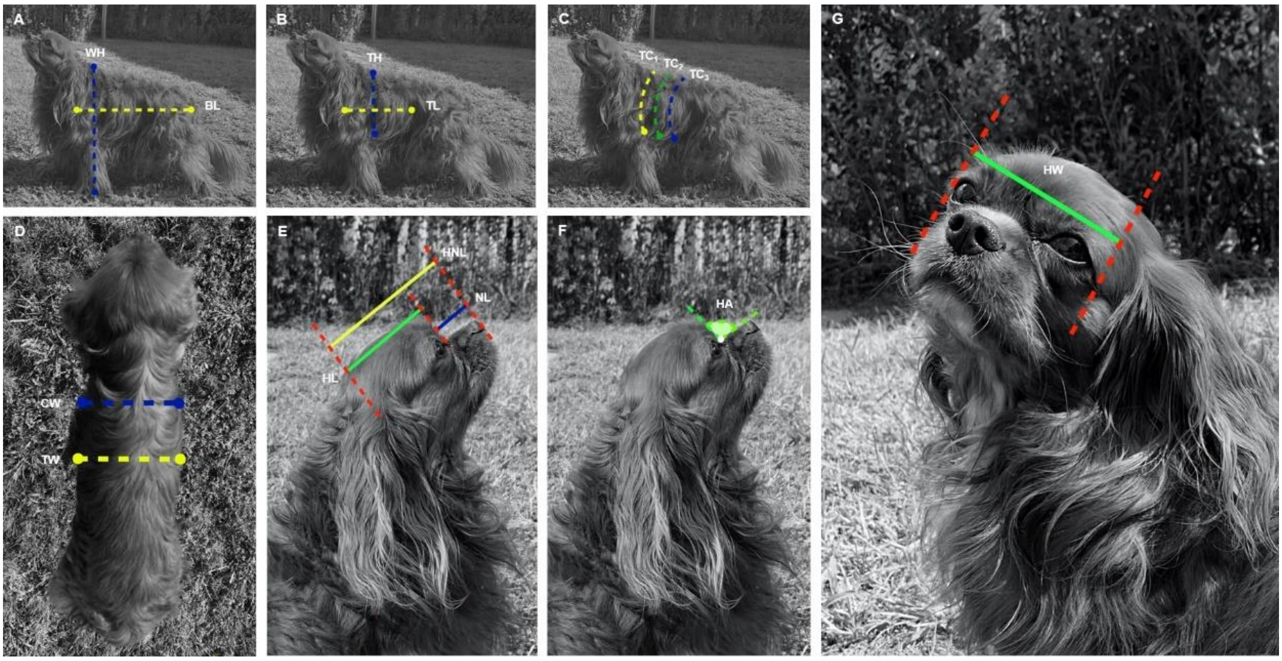


**Figure S1.** Demonstration of the instrumental and soft-tape measurements. Notes: Body and thorax dimensions were measured using a custom-made sliding gauge. Thoracic circumferences were measured while the dog was standing and at rest with a firmly held soft tape measure. Head length, nose length, head and nose length, and head width were measured using a gauge. A goniometer was used to measure the head's stop angle. The detailed definitions of the measurements are shown in Table 1 [33]. The dogs represented here were included in the present study and photographed by the authors. (**A**) WH = height at the withers (cm) (blue line); BL = body length (cm) (yellow line). (**B**) TH = thorax height (cm) (blue line); TL = thorax length (cm) (yellow line). (**C**) TC1 = thoracic anterior or axillary circumference (cm) (yellow line); TC2 = thoracic mean or papillary circumference (cm) (green line); TC3 = thoracic lower or basal circumference (cm) (blue line). (**D**) CW = chest width (cm) (blue line); TW = thorax width (cm) (yellow line). (**E**) HL = head length (cm) (green line); NL = nose length (cm) (blue line); HNL = head-nose length (cm) (yellow line). (**F**) HA = head stop angle (°) (green). (**G**) HW = head width (cm) (red line).
